# Preference, Expected Burden, and Willingness to Use Digital and Traditional Methods to Assess Food and Alcohol Intake

**DOI:** 10.3390/nu13103340

**Published:** 2021-09-24

**Authors:** Christoph Höchsmann, Nicole Fearnbach, James L. Dorling, Tera L. Fazzino, Candice A. Myers, John W. Apolzan, Corby K. Martin

**Affiliations:** 1 Pennington Biomedical Research Center, Baton Rouge, LA 70808, USA; hoechsmann.christoph@gmail.com (C.H.); nicole.fearnbach@pbrc.edu (N.F.); james.dorling@glasgow.ac.uk (J.L.D.); candice.myers@pbrc.edu (C.A.M.); john.apolzan@pbrc.edu (J.W.A.); 2 Cofrin Logan Center for Addiction Research and Treatment, University of Kansas, Lawrence, KS 66045, USA; tfazzino@ku.edu; 3 Department of Psychology, University of Kansas, Lawrence, KS 66045, USA

**Keywords:** food intake, food records, RFPM, PortionSize, diet recall, alcohol

## Abstract

We conducted an online survey to examine the preference, expected burden, and willingness of people to use four different methods of assessing food and alcohol intake such as food/drink record, 24-h recall, Remote Food Photography Method© (RFPM, via SmartIntake^®^ app), and a novel app (PortionSize^®^) that allows the in-app portion size estimation of foods/drinks by the user. For food (N = 1959) and alcohol (N = 466) intake assessment, 67.3% and 63.3%, respectively, preferred the RFPM/SmartIntake^®^, 51.9% and 53.4% preferred PortionSize^®^, 48.0% and 49.3% the food records, and 32.9% and 33.9% the 24-h recalls (difference in preference across all methods was *p* < 0.001 for food and alcohol intake). Ratings of burden and preference of methods were virtually superimposable, and we found strong correlations between high preference and low expected burden for all methods (all ρ ≥ 0.82; all *p* < 0.001). Willingness (mean (SD)) to use the RFPM/SmartIntake^®^ (food: 6.6 (2.0); alcohol: 6.4 (2.4)) was greater than PortionSize^®^ (food: 6.0 (2.2); alcohol: 6.0 (2.4); all *p* < 0.001) and 24-h recalls (food: 6.1 (2.2); alcohol: 5.7 (2.7); *p* < 0.001), but not different from food records (food: 6.6 (2.0); alcohol: 6.5 (2.3); all *p* ≥ 0.33). Our results can be used in conjunction with existing data on the reliability and validity of these methods in order to inform the selection of methods for the assessment of food and alcohol intake.

## 1. Introduction

Several factors affect the suitability of different methods of ingestive behavior assessment in specific study designs, clinical settings, and populations. Validity, reproducibility, usability, and feasibility of the selected method in the target population and data collection setting are indispensable for the collection of high-quality data on ingestive behavior. However, participants’ preference as well as the acceptability and perceived burden of a specific method can also play an important role in collecting high-quality data.

It has been reported that adherence to more burdensome self-report methods of assessing dietary intake can be low and typically decreases over time, thereby influencing data quality [1,2,3]. Furthermore, pen-and-paper food checklists to track food and beverage intake have been reported to be preferred (46% of participants) and perceived as less burdensome compared to both 24-h recalls (29%) and pen-and-paper food records with the additional requirement of weighing all foods consumed (21%) [4]. In a cross-over study, 78% of participants preferred using the online version of a food record compared to 13% who preferred the pen-and-paper record (9% had no preference) after having used each method for 7 days. The online method was perceived as being quicker, more convenient, and overall less burdensome than the paper version [5]. Collecting data online or via an app offers some additional advantages compared to pen-and-paper methods, including the ability for data to be transferred in real time.

Some ingestive behavior assessment methods attempt to reduce participant burden and improve accuracy by asking participants to capture images of food and drinks with camera-enabled devices such as smartphones, and these images are then analyzed by a trained rater, not the participant, to estimate intake. One such method is called the Remote Food Photography Method© (RFPM), which involves participants capturing images of food and drinks and annotating the images with descriptors in order to identify products that are not readily identifiable by wrappers or logos in the image [6,7]. These images are then sent to researchers or clinicians for analysis in near-real time. The collection of RFPM data is streamlined by a custom-built smartphone app called SmartIntake^®^, which participants use to capture images of their food/drink selection and plate/drink waste with a smartphone or tablet; the app automatically sends the food images and related data wirelessly to the laboratory for analysis [6,7]. This reduces errors in portion size estimation [6], the largest source of error in self-reported food intake [8]; more importantly, because the burden of estimating portion size is moved from the participant to the researcher or clinician, this may help explain the large difference in preference for this method compared to traditional self-report methods. For example, the RFPM/SmartIntake^®^ has been found to be preferred by 93.6% of participants for assessing food intake compared to pen-and-paper records [7], and 93.3% of participants preferred the RFPM/SmartIntake^®^ compared to online diet recalls for assessing alcohol consumption [9] after using each method for 3 consecutive days in free-living conditions. However, the preference for app-based methods that rely on users to estimate portion size from food images has not been evaluated.

To date, a direct comparison of the perceived burden and preference across these methods (food/drink record, 24-h recall, RFPM) that are commonly used to assess ingestive behavior is lacking. The assumption that app- and image-based methods are perceived as less burdensome and consequently preferred compared to traditional self-report methods (food/drink record, 24-h recall) has not been thoroughly examined. To address this, we conducted an online survey to assess participants’ preference, expected burden, and willingness to use four different methods of food/drink intake assessment: (1) a food/drink record; (2) a 24-h recall; (3) the RFPM/SmartIntake^®^ app; and (4) a novel smartphone app (PortionSize^®^) that allows the estimation of the portion size of foods and drinks by the user directly in the app, which results in immediate food and drink intake feedback without the need for external analysis by a trained clinician or researcher. We hypothesized that for both food intake and alcohol consumption, the RFPM/SmartIntake^®^ and PortionSize^®^ would be rated as more preferred than the traditional methods (food record and 24-h recall) and that the RFPM/SmartIntake^®^ would additionally be perceived as less burdensome and more preferred compared to PortionSize^®^, which requires participants to estimate and report their portion size.

## 2. Materials and Methods

### 2.1. Design and Participants

The Pennington Habits Survey was approved by the Institutional Review Board at Pennington Biomedical Research Center (PBRC, 2019-052-PBRC) and registered at ClinicalTrials.gov (NCT04150510) before the start of recruitment. The survey included a questionnaire assessing demographic and socioeconomic characteristics as well as: (1) the Food Intake Assessment Preference Questionnaire, (2) the Alcohol Consumption Questionnaire, (3) Alcohol Consumption Assessment Preference Questionnaire, (4) the Smoking Questionnaire, (5) the Smoking Assessment Preference Questionnaire, (6) the Vaping Questionnaire, and (7) the Vaping Assessment Preference Questionnaire. A link to the anonymous survey was distributed by paid advertisements on social media platforms, PBRC’s webpage, email listservs, and word-of-mouth between February and November 2020. Adults between 18 and 85 years of age, residing in the United States, and with access to the internet were eligible to participate in the Pennington Habits Survey. However, to complete the Assessment Preference Questionnaires for alcohol consumption, smoking, and vaping, participants had to indicate that they were practicing the respective activities at the time of the survey. Upon opening the survey link, interested individuals received instructions that detailed the purpose of the study. Participants verified that they were adults and provided consent to participate before proceeding with the survey. Data were collected using Research Electronic Data Capture (REDCap) [10]. Participation in the survey was voluntary and the participants had the option not to submit answers or to skip items if they did not wish to complete them. Upon completion of the survey, participants had the option to enter a lottery to win 1 of 10 checks worth USD 50. In this report, only the results from the Food Intake Assessment Preference Questionnaire and the Alcohol Consumption Assessment Preference Questionnaire as well as associated demographic data are presented.

### 2.2. Demographic and Socioeconomic Characteristics

Before continuing with the Food Intake and Alcohol Consumption Assessment Preference Questionnaires, participants completed a questionnaire assessing demographic and socioeconomic characteristics. The questionnaire captured data such as age, sex, race, education level, and household income. In addition, household food security (assessed with the 6-item Short Form of the United States Household Food Security Survey Module) [11] and subjective social status (assessed with the MacArthur Scale of Subjective Social Status, in which individuals place an “X” on the rung (1–10) of the “social ladder” on which they feel they stand compared to other people in the United States) [12] were assessed. Finally, participants self-reported body weight and height, and past or present diagnosis of one or more of the following diseases: heart disease, type 2 diabetes, hypertension, or dyslipidemia.

### 2.3. Food Intake and Alcohol Consumption Assessment Preference Questionnaires

Participants were provided with a description of the following 4 methods of food intake and alcohol consumption assessment: (1) a food/drink record; (2) a 24 h recall; (3) the RFPM and SmartIntake^®^ app [7], which has also been used to assess alcohol consumption [9]; and (4) a new smartphone app called PortionSize^®^. PortionSize^®^ provides immediate food and beverage intake feedback to the participant and researchers since the participant estimates portion size in the app based on their food/drink images without the need for external analysis by a trained clinician or researcher. The verbatim descriptions and images, if applicable, for the 4 methods of food intake assessment that were provided to the participants in the survey are outlined below and also provided as Supplementary Methods. The descriptions for the 4 methods of alcohol consumption assessment were very similar.

#### 2.3.1. Food Record

“Food records are a way to record all of the foods and beverages that you consume. You are usually asked to keep these records for 3–7 days. During this period, you would need to carry the record with you and record all foods and beverages that you consume right when you eat or drink them. The food record can be a paper form that you complete by hand. Other ways to keep these records include using a smartphone to complete the record electronically. To increase the accuracy of the record, you need to carefully estimate or weigh how much food you eat, and how many beverages you drink and record those amounts. Additionally, you need to record details about the food or beverage. Those details include things like what cut of meat you are eating, what condiments you added, and how much condiments you added. Finally, you need to record how the food was cooked or prepared. For example, if the food was fried, baked, sautéed, etc.”

#### 2.3.2. The 24-h Recall

“A 24 h recall is another method to track your food and beverage intake. This method is like an interview that is usually conducted via phone or in person. Each interview takes 20 to 30 min. You would be asked to recall all of the foods and beverages that you consumed over the previous 24 h. You also would need to recall and report how much of each food and beverage you consumed. Finally, you would need to recall and report how the foods were prepared (fried, baked, etc.). Because our food intake varies from day to day, you would typically be asked to complete about 3 of these interviews.”

#### 2.3.3. RFPM/SmartIntake^®^

“Smartphone-based methods can record food intake based on pictures of food that you capture with a smartphone app. Specifically, you would use an app to take pictures of your meals before and after you eat. If it is not clear what you are eating or drinking, you would type in a brief description of those foods. The app then automatically sends the pictures and information you entered to nutrition professionals. Those nutrition professionals can then estimate how much you ate and drank based on the pictures. The app also automatically reminds you to capture images of your meals. Those reminders are customized based on your schedule and eating habits.”

#### 2.3.4. PortionSize^®^

**Figure 1:**
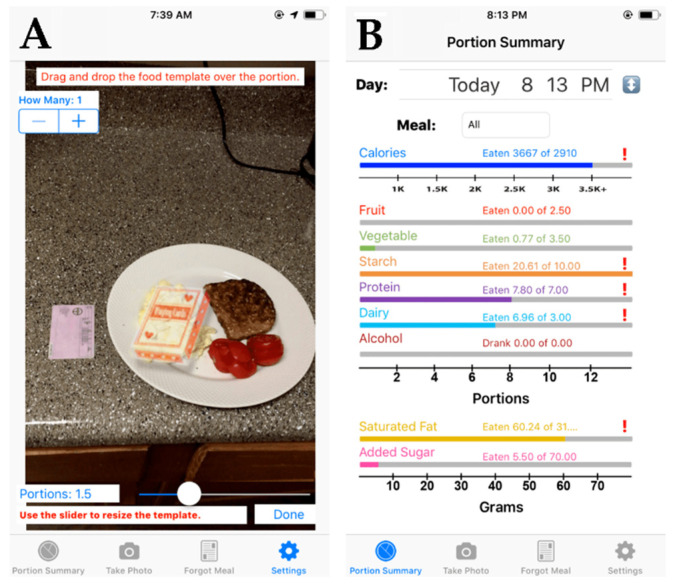
Screenshots from the PortionSize^®^ app illustrating an example in which a template that looks like a deck of cards was placed over scrambled eggs to estimate their portion size (**A**) as well as an example of the real-time feedback (calories and nut and nutrient composition of the meal and the overall diet) provided by the PortionSize^®^ app after the automatic portion size estimation (**B**).

“More recently, smartphone apps have been developed that do not require the analysis of the food images by a nutrition professional. Rather, you would estimate the portion size of the foods directly in the app. You would take a picture of your meal before you ate. You would then identify the foods and beverages in the meal via a drop-down list or with a search function. To estimate the portion size of the foods in the picture, you would do one of two things. First, you can enter in the amount of all food(s) consumed or the size of the food if it is known (e.g., 4 Famous Amos cookies, one 12-ounce regular Coke). Second, you can use templates that appear in the app. You can adjust the size of these templates and move them within the picture of the foods. Hence, to estimate portion size, you would change the size of the template and move it so it covers the food. Figure 1A illustrates an example, in which a template that looks like a deck of cards was placed over scrambled eggs. The app then uses this information to automatically and immediately estimate how much food is on your plate. After the meal, you enter if you ate everything, left a certain amount of the food on your plate, or you can use the templates again to estimate large portions of leftovers. This allows the app to provide a more accurate estimate of how much you ate. The app gives you real-time feedback about how many calories you ate and the nutrient composition of your meal and overall diet.” An example of that feedback is provided in Figure 1B.

### 2.4. Measures

#### 2.4.1. Preference of Methods

Participants ranked the 4 methods for food intake and alcohol consumption from most to least preferred, assuming they would use each method to record their food intake/alcohol consumption for 3 days as part of a clinical or study setting. The timeframe of 3 days is commonly used in clinical studies that aim to assess food and (alcoholic) drink intake [9,13,14,15].

#### 2.4.2. Expected Burden of Methods

Similar to the preference of methods, participants further ranked the 4 methods for food intake and alcohol consumption from least to most burdensome according to their expected burden, assuming they would use each method to record food intake/alcohol consumption for 3 days as part of a clinical or study setting.

#### 2.4.3. Willingness to Use Methods

Furthermore, participants rated their willingness to use a food/drink record, the RFPM/SmartIntake^®^, and PortionSize^®^ to record their food intake and alcohol consumption over a period of 3 days on an 8-point Likert scale from ‘not at all’ (rated as 1) to ‘very much’ (rated as 8). For the 24 h recall, participants rated their willingness to complete 3 separate recall interviews on the same Likert scale.

### 2.5. Statistical Analyses

Data were cross-sectional and analyzed descriptively. We present categorical variables as frequency (%) and continuous data, including Likert scale items, as mean (standard deviation (SD)). For the main analysis, to simplify the presentation of results and allow for a natural binary comparison, we categorized the ‘most preferred’ (1st choice) and ‘second-most preferred’ (2nd choice) as ‘preferred’, and similarly, the ‘second-least preferred’ (3rd choice) and ‘least preferred’ (4th choice) as ‘not preferred’, in addition to the individual ranks (1st through 4th choice). We examined differences between ‘preferred’ and ‘not preferred’ for each method as well as differences in ratings across methods with chi-square statistics. In additional exploratory subgroup analyses, we assessed the effect of age (categories: <25 years, 25–34 years, 35–44 years, 45–54 years, 55–64 years, and ≥65 years), sex (male vs. female), race (categories: White, Black, Native American, Asian or Pacific Islander, and other), education (categories: less than high school, high school or equivalent, bachelor’s degree, master’s degree, doctorate, and other), household income (categories: <USD 10,000, USD 10,000–50,000, USD 50,000–100,000, USD 100,000–150,000, and >USD 150,000), household food security (categories: high food security, low food security, and very low food security), subjective social status, BMI (categories: <25 kg/m^2^, 25.0–29.9 kg/m^2^, and ≥30.0 kg/m^2^, calculated from self-reported height and weight), and cardiometabolic diseases (positive past or present diagnosis vs. no diagnosis) on the preference of methods. For the expected burden of methods, similar to the preference of methods, we categorized the ‘least burdensome’ (1st choice) and ‘second-least burdensome’ (2nd choice) as ‘low expected burden’, and the ‘second-most burdensome’ (3rd choice) and ‘most burdensome’ (4th choice) as ‘high expected burden’, in addition to the individual ranks (1st through 4th choice). We examined differences between ‘low expected burden’ and ‘high expected burden’ for each method as well as differences in ratings across methods with chi-square analyses and ran the same subgroup analyses as for the preference of methods. Correlations between preference (1st through 4th choice) and expected burden (1st through 4th choice) of methods for food intake as well as alcohol consumption were analyzed using Spearman’s rank correlation coefficient. Differences in willingness to use each method to monitor food intake and alcohol consumption over 3 days were assessed by analysis of covariance (ANCOVA), and we used a Tukey adjustment for post hoc pairwise comparisons. Differences in willingness as examined by the Kruskal–Wallis test did not differ meaningfully (not reported). All analyses were conducted in SPSS version 25. Due to multiple comparisons, the significance level was set to 0.001.

## 3. Results

### 3.1. Participant Characteristics

A total of 3245 adults participated in the online Pennington Habits Survey, and 1959 participants completed the Food Intake Assessment Preference Survey and are included in the main analyses. Participant characteristics are provided in Table 1. On average, participants (78.5% women, 78.2% White) were 45.9 (SD: 16.4) years old and had a BMI of 30.8 (SD: 8.8), with the majority of participants having either overweight (26.6%) or obesity (45.0%). Most participants indicated a high school diploma or equivalent (29.9%) or bachelor’s degree (34.7%) as their highest level of education, and two-thirds reported a household income between USD 10,000 and USD 100,000. Three-quarters of the participants reported high household food security, and 64.8% of the participants saw themselves on rung 5, 6, or 7 of the MacArthur Scale of Subjective Social Status.

A subsample of 466 participants reported consuming alcohol at the time of the survey and completed the Alcohol Consumption Assessment Preference Questionnaire. Characteristics of those participants are provided in Table 2. On average, participants had been consuming alcohol regularly for 26.5 (SD: 15.9) years. Three-quarters of the participants reported drinking no more than 1–2 times per week (34.3%) or only on special occasions (40.3%), and 86.5% of the participants reported drinking ≤ 3 drinks per typical drinking occasion.

### 3.2. Methods of Food Intake Assessment

#### 3.2.1. Preference of Methods

**Figure 2:**
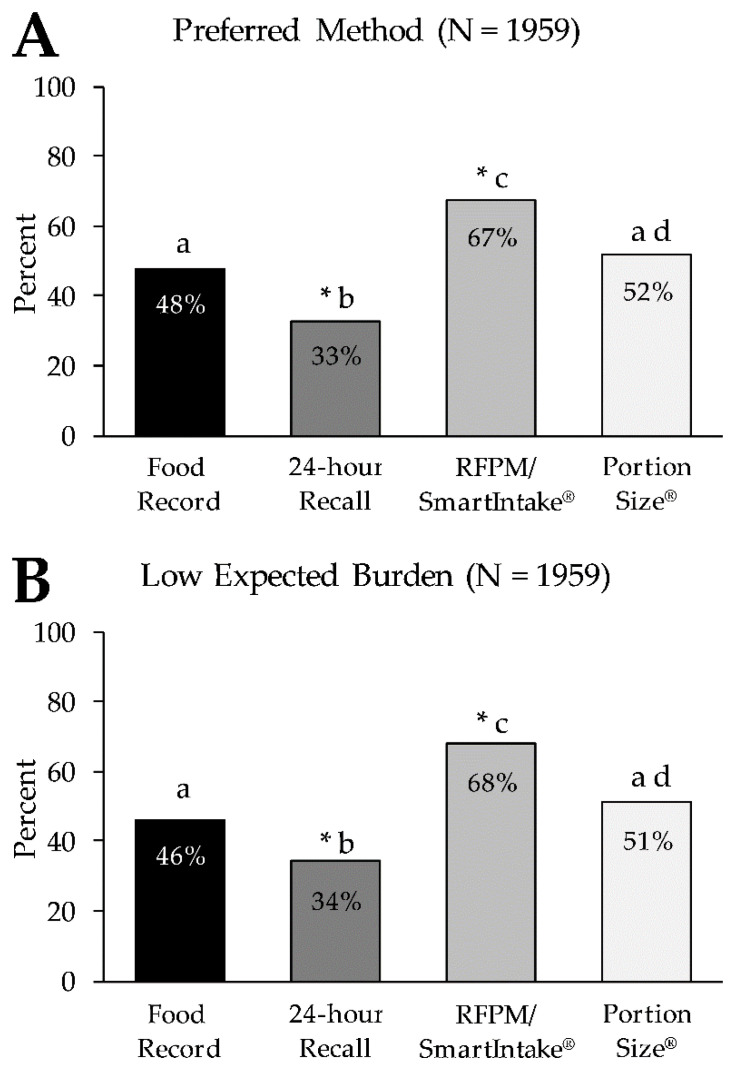
Percentage of participants who rated each method as their preferred method of food intake assessment (Panel **A**) and who rated the expected burden of each method as low (Panel **B**). * Denotes a significant difference in ‘not preferred’ (Panel **A**) and in ‘high expected burden’ (Panel **B**) for the respective method (*p* < 0.001) in the same panel. Letters (a–d) that differ from each other indicate differences between methods (*p* < 0.001) in the same panel. RFPM, Remote Food Photography Method.

Figure 2A shows the percentage of participants who rated each method as their preferred method of food intake assessment. The RFPM/SmartIntake^®^ was rated as the preferred method by the largest percentage of participants (67.3%), while the 24 h recall was rated as the preferred method by the smallest percentage of participants (32.9%). The food record and PortionSize^®^ were rated as the preferred method by 48.0% and 51.9%, respectively, with an overall difference in preference across all methods (*p* < 0.001). Pairwise comparisons showed differences in preference ratings between all methods (*p* < 0.001), except between the food record and PortionSize^®^ (*p* = 0.06). Table 3 displays the frequencies (%) for the individual ranks (1st through 4th choice) for the different methods. There was an overall difference in preference ratings across all methods (*p* < 0.001), and pairwise comparisons showed differences between all methods (*p* < 0.001), except between the food record and PortionSize^®^ (*p* = 0.35).

**Figure 3:**
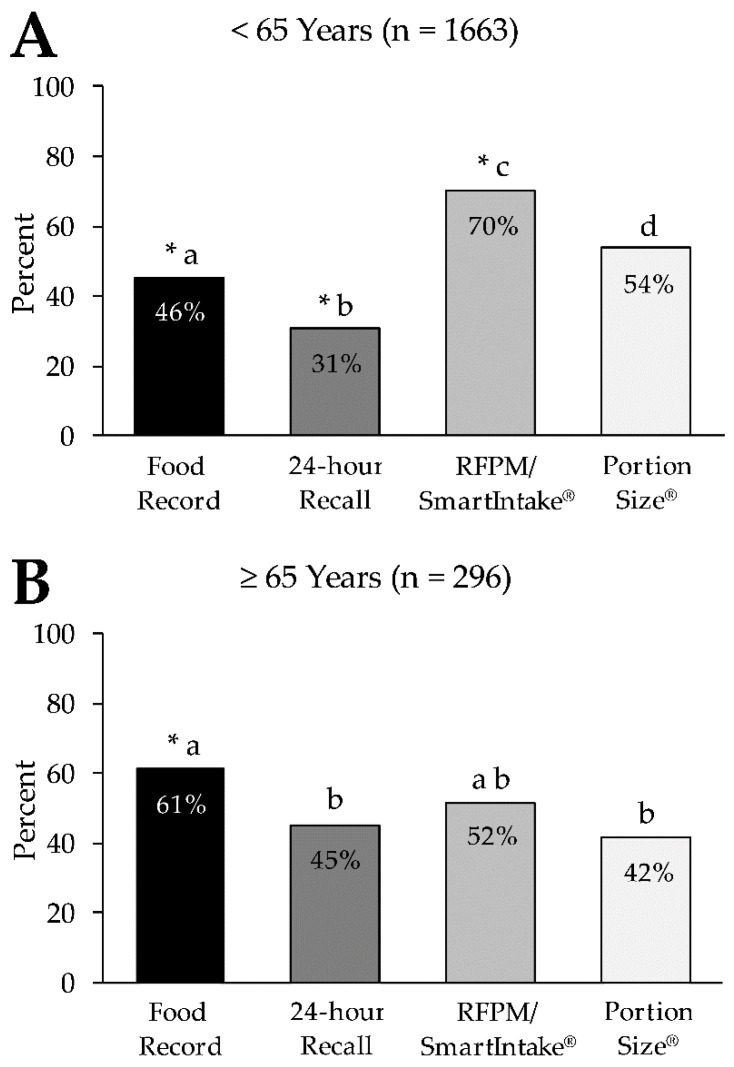
Percentage of participants who rated each method as their preferred method of food intake assessment in those <65 years (Panel **A**) and those ≥65 years of age (Panel **B**). * Denotes a significant difference in ‘not preferred’ for the respective method (*p* < 0.001) in the same panel. Letters (a–d) that differ from each other indicate differences between methods (*p* < 0.001) in the same panel. RFPM, Remote Food Photography Method.

We found a significant age effect on the preference ratings of the four methods (*p* < 0.001). This effect was primarily driven by the difference between those <65 years and those ≥65 years of age (Figure 3). A greater percentage of participants ≥65 years rated the food record (61.4%) and 24 h recall (45.3%) as a preferred method compared to participants <65 years (food record: 45.5%, 24 h recall: 30.6%, all *p* < 0.001). Conversely, a greater percentage of participants <65 years rated the RFPM/SmartIntake^®^ (70.1%) and PortionSize^®^ (53.7%) as their preferred method compared to those ≥65 years (RFPM/SmartIntake^®^: 51.7%, PortionSize^®^: 41.6%, all *p* < 0.001). Supplementary Figure S1 shows the preference ratings of the four methods for all age categories. Differences in ratings of the four methods between two age categories were only significant when compared with those ≥65 years, with the single exception of PortionSize^®^, which had a significantly higher preference rating in those aged 35–44 (61.2%) compared to those aged 25–34 (46.7%, *p* < 0.001). All other comparisons of methods across age categories were not significant (all *p* ≥ 0.05).

We also found a significant disease effect on the preference ratings of the four methods (*p* = 0.001). However, pairwise comparisons of the preference ratings of the four methods between those with a past or present diagnosis of cardiometabolic disease and those with no such diagnosis were all not significant (all *p* ≥ 0.06). We did not find any effects on the preference ratings for the four methods by sex, race, education level, household income, household food security, subjective social status, or BMI (all *p* ≥ 0.07; data not shown).

#### 3.2.2. Expected Burden of Methods

Figure 2B shows the percentage of participants who rated the expected burden of each method of food intake assessment as low. The largest percentage of participants (68.1%) rated the expected burden of the RFPM/SmartIntake^®^ as low, followed by PortionSize^®^ (51.4%), the food record (46.2%), and the 24 h recall (34.3%), with an overall difference in expected burden across all methods (*p* < 0.001). Pairwise comparisons showed differences in preference ratings between all methods (*p* < 0.001), except between the food record and PortionSize^®^ (*p =* 0.06). Table 3 displays the frequencies (%) for the individual ranks (1st through 4th choice) for the different methods. There was an overall difference in preference ratings across all methods (*p* < 0.001), and pairwise comparisons showed differences between all methods (*p* < 0.001), except between the food record and PortionSize^®^ (*p =* 0.96).

Similar to the preference of methods, we found a significant age effect on ratings of the expected burden of the four methods (*p* < 0.001), and this effect appeared to be driven by the difference between those <65 years and those ≥65 years of age. A greater percentage of participants ≥65 years rated the expected burden of the food record (61.1%) and 24 h recall (46.6%) as low compared to participants <65 years (food record: 43.6%, 24 h recall: 32.0%, all *p* < 0.001). Conversely, a greater percentage of participants <65 years rated the expected burden of the RFPM/SmartIntake^®^ (71.3%) and PortionSize^®^ (53.1%) as low compared to those ≥65 years (RFPM/SmartIntake^®^: 50.3%, PortionSize^®^: 41.9%, all *p* < 0.001). Differences in ratings of the expected burden of the four methods between all other age categories were only significant when compared with those ≥65 years. All other comparisons of methods across age categories were not significant (all *p* ≥ 0.07). We did not find any effects on preference ratings for the four methods by sex, race, education level, household income, household food security, subjective social status, BMI, or diagnosis of cardiometabolic disease (all *p* ≥ 0.06; data not shown).

#### 3.2.3. Correlation between Preference and Expected Burden of Methods

High preference was strongly correlated with low expected burden of the respective method for all methods of food intake assessment, with coefficients of ρ *=* 0.85 (*p* < 0.001) for the food record, ρ *=* 0.85 (*p* < 0.001) for the 24 h recall, ρ *=* 0.82 (*p* < 0.001) for the RFPM/SmartIntake^®^, and ρ *=* 0.85 (*p* < 0.001) for PortionSize^®^.

#### 3.2.4. Willingness to Use Methods

The willingness to use the method over 3 days to monitor food intake was rated (8-point Likert scale) with a mean of 6.6 (SD: 2.0) for the food record, 6.1 (SD: 2.2) for the 24 h recall, 6.6 (SD: 2.0) for the RFPM/SmartIntake^®^, and 6.0 (SD: 2.2) for PortionSize^®^ (Table 3), with a significant main effect (*p* < 0.001). Post hoc comparisons showed that willingness to use the food record differed from the 24 h recall (*p* < 0.001) and PortionSize^®^ (*p* < 0.001) but not from the RFPM/SmartIntake^®^ (*p* = 0.96). Willingness to use the 24 h recall differed from the RFPM/SmartIntake^®^ (*p* < 0.001) but not from PortionSize^®^ (*p* = 0.23), and willingness to use the RFPM/SmartIntake^®^ was greater than PortionSize^®^ (*p* < 0.001).

### 3.3. Preference of Methods for Alcohol Consumption Assessment

#### 3.3.1. Preference of Methods

**Figure 4:**
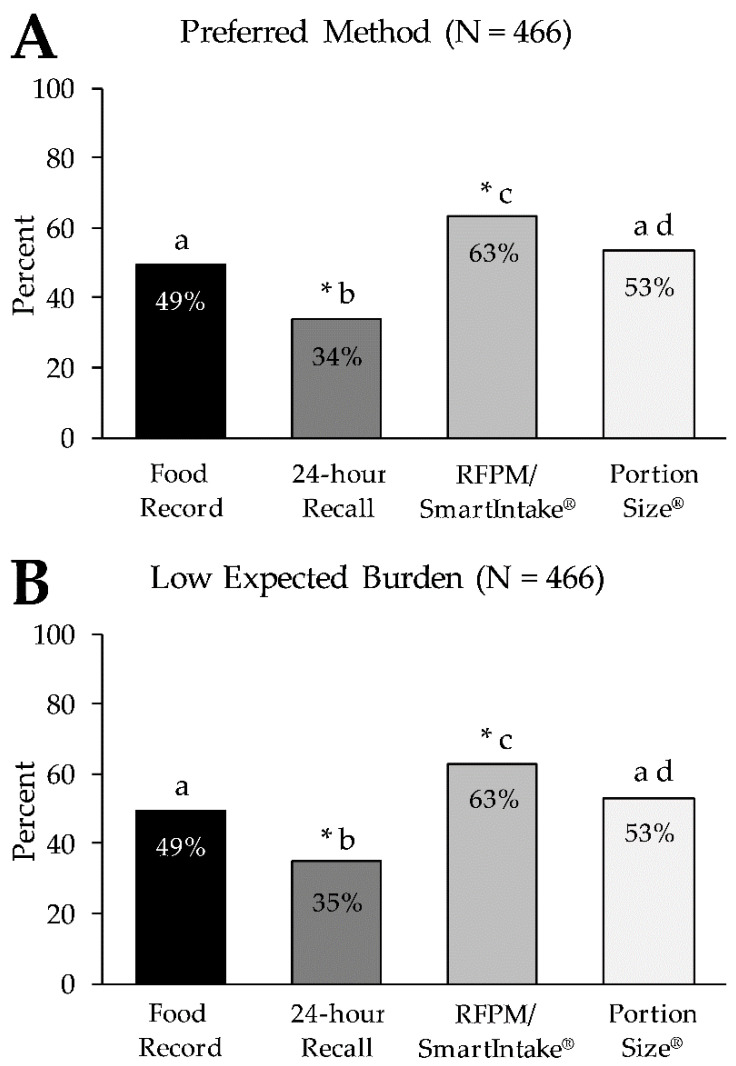
Percentage of participants who rated each method as their preferred method of alcohol consumption assessment (Panel **A**) and who rated the expected burden of each method as low (Panel **B**). * Denotes a significant difference in ‘not preferred’ for the respective method (*p* < 0.001) in the same panel. Letters (a–d) that differ from each other indicate differences between methods (*p* < 0.001) in the same panel. RFPM, Remote Food Photography Method.

Figure 4A shows the percentage of participants who rated each method as their preferred method of alcohol consumption assessment. Similar to the food intake assessment, the RFPM/SmartIntake^®^ was rated as the preferred method by the largest percentage of participants (63.3%), while the 24 h recall was rated as the preferred method by the smallest percentage of participants (33.9%). The food record and PortionSize^®^ were rated as the preferred method by 49.3% and 53.4% respectively, with an overall difference in preference across all methods (*p* < 0.001). Pairwise comparisons showed differences in preference ratings between all methods (*p* < 0.001), except between the food record and PortionSize^®^ (*p* = 0.34). Table 4 displays the frequencies (%) for the individual ranks (1st through 4th choice) for the different methods. There was an overall difference in preference ratings across all methods (*p* < 0.001), and pairwise comparisons showed differences between all methods (*p* < 0.001), except between the food record and PortionSize^®^ (*p =* 0.26). We did not find any effects on preference ratings for the four methods by sex, age, race, education level, household income, household food security, subjective social status, BMI, or diagnosis of cardiometabolic disease (all *p* ≥ 0.13).

#### 3.3.2. Expected Burden of Methods

Figure 4B shows the percentage of participants who rated the expected burden of each method of alcohol consumption assessment as low. Similar to the food intake assessment, the largest percentage of participants (63.1%) rated the expected burden of the RFPM/SmartIntake^®^ as low, followed by PortionSize^®^ (52.8%), the food record (49.4%), and the 24 h recall (34.7%), with an overall difference in expected burden across all methods (*p* < 0.001). Pairwise comparisons showed differences in the ratings of expected burden between all methods (*p* < 0.001), except between the food record and PortionSize^®^ (*p* = 0.43). Table 4 displays the frequencies (%) for the individual ranks for the different methods. There was an overall difference in preference ratings across all methods (*p* < 0.001), and pairwise comparisons showed differences between all methods (*p* < 0.001), except between the food record and PortionSize^®^ (*p* = 0.23). We did not find any effects on the ratings of the expected burden of the four methods by sex, age, race, education level, household income, household food security, subjective social status, BMI, or diagnosis of cardiometabolic disease (all *p* ≥ 0.06).

#### 3.3.3. Correlation between Preference and Expected Burden of Methods

Similar to food intake, high preference was strongly correlated with a low expected burden of the respective method for all methods of alcohol consumption assessment with coefficients of ρ *=* 0.91 (*p* < 0.001) for the food record, ρ *=* 0.89 (*p* < 0.001) for the 24 h recall, ρ *=* 0.90 (*p* < 0.001) for the RFPM/SmartIntake^®^, and ρ *=* 0.91 (*p* < 0.001) for PortionSize^®^.

#### 3.3.4. Willingness to Use Methods

Willingness to use each method over 3 days to monitor alcohol consumption was an average of 6.5 (SD: 2.3) for the food record, 5.7 (SD: 2.7) for the 24 h recall, 6.4 (SD: 2.4) for the RFPM/SmartIntake^®^, and 6.0 (SD: 2.4) for PortionSize^®^ (Table 4), with a significant main effect (*p* < 0.001). Post hoc comparisons showed that the willingness to use the food record differed from the 24 h recall and PortionSize^®^ (all *p* < 0.001) but not from the RFPM/SmartIntake^®^ (*p* = 0.33). Willingness to use the 24 h recall differed from the RFPM/SmartIntake^®^ (*p* < 0.001) but not from PortionSize^®^ (*p =* 0.50), and willingness to use the RFPM/SmartIntake^®^ was greater than PortionSize^®^ (*p* < 0.001).

## 4. Discussion

The present study used an online survey to assess the preference for and the expected burden of four different methods of food intake and alcohol consumption assessment (food record, 24-h recall, RFPM/SmartIntake^®^, PortionSize^®^) as well as the willingness to use these methods to record food intake and alcohol consumption over 3 days.

In line with our hypotheses for both food intake and alcohol consumption assessment, the RFPM/SmartIntake^®^ was rated more preferred and less burdensome than more traditional methods (food record and 24-h recall), which is consistent with previous findings [7,9]. Additionally, the RFPM/SmartIntake^®^ was perceived as less burdensome and more preferred compared to PortionSize^®^, as hypothesized. As illustrated in Figure 2; Figure 4, the graphs depicting the preference for and expected burden of the methods are virtually superimposable, and the strong correlations between high preference and low expected burden for all methods (ρ ≥ 0.82; *p* < 0.001) support the hypothesis that expected burden influences method preference. It has previously been reported that more burdensome methods yield low adherence [1,2,3]. It is conceivable that methods that are more preferred by participants (due to lower perceived burden) lead to better adherence and consequently greater data quality over a longer period of time (or during repeated assessment periods) compared to less preferred methods. The difference in ratings of expected burden between the RFPM/SmartIntake^®^ and PortionSize^®^ could mean that the participants recognized that the self-estimation of the portion size of foods in PortionSize^®^ would require more effort on their part, while this burden is shifted to a researcher or clinician when using RFPM/SmartIntake^®^. Furthermore, our hypothesis that PortionSize^®^ would be rated more preferred than the traditional methods was only partially supported, as PortionSize^®^ was only rated more preferred and less burdensome compared to the 24-h recall but not to the food record. This suggests that while mHealth technology certainly holds promise in reducing the burden of dietary self-monitoring [16,17], some mHealth methods are more burdensome than others, and differences in burden are detected by prospective users. Additionally, some mHealth methods are likely to be perceived as more burdensome in relation to streamlined self-report methods such as a checklist associated with a structured meal plan. The overall low preference along with the relatively high expected burden of the 24-h recall is somewhat surprising, particularly when compared to the food record. The 24-h recall, as described to participants, requires three relatively brief (20–30 min) recall interviews, whose de facto time burden is likely less than keeping a detailed food record for 3–7 days [18], which in many studies requires participants to log every eating occasion, preferably in real time and as comprehensively as possible, in order to improve weight loss outcomes [19,20]. It can be conjectured that other factors of the 24-h recall were unappealing to participants, such as the interview format, and that this influenced the ratings of burden and preference for that method. Additionally, participants did not use each method, and actual experience with the methods could influence the ratings.

The preference for and expected burden of methods was independent of sex, race, education level, household income, household food security, subjective social status, BMI, or diagnosis of cardiometabolic disease for both food intake and alcohol consumption assessment. However, we must acknowledge that our participants were predominantly women (78.5%), White (78.2%), food secure (74.0%), with overweight or obesity (71.6%), and had a college education (60.4%). A more heterogeneous sample might have led to different results. Age affected the preference ratings of the methods for assessing food intake but not alcohol consumption. While there was a clear pattern in those <65 years with RFPM/SmartIntake^®^ > PortionSize^®^ > food record > 24-h recall (from the most to the least preferred), in those ≥65 years, preferences were more evenly distributed across the four methods. The food record (61.4%) was the method that was preferred by the largest percentage in those ≥65 years, followed by the RFPM/SmartIntake^®^ (51.7%; not different from food record). The 24-h recall (45.3%) and PortionSize^®^ (41.6%; no difference between the 2 methods) were less preferred compared to the food record. A recent cross-sectional study (N *=* 364) that assessed older adults’ intention to use mHealth apps showed that 49.7% of the participants (mean age 75 (SD: 7) years) had no intention to use any such apps [21], which might explain why participants ≥65 in our study rated app-based methods (RFPM/SmartIntake^®^, PortionSize^®^) as relatively less preferred than traditional methods (food record, 24-h recall) compared to participants <65 years. However, while the food record was the most preferred method to assess food intake among those ≥65 years, preference for the RFPM/SmartIntake^®^ did not differ significantly and more than half of those ≥65 years still rated the RFPM/SmartIntake^®^ as their preferred method. The RFPM/SmartIntake^®^ is accurate and more accurate than food records [6,9,22]; hence, the RFPM/SmartIntake^®^ remains a viable method for participants ≥65 years of age. Nevertheless, the ability of older adults to reliably use apps needs to be evaluated before data collection. Additional support from the study staff and an easy-to-use interface of the apps may help overcome potential barriers of app-based methods and increase the acceptability and comfort of these technologies [23], which would ensure reliable use and high-quality data collection throughout the study period.

Furthermore, and in line with the overall findings on preference and expected burden of methods, the willingness to use the RFPM/SmartIntake^®^ was greater than that for the 24-h recall and PortionSize^®^ for assessing both food and alcohol intake. However, despite the overall greater preference and lower expected burden of the RFPM/SmartIntake^®^ compared to the food record, the willingness to use each method for 3 days did not differ. This is interesting and suggests that while a low expected burden of a method is strongly correlated with a high preference for that method, the willingness to use the method to record food intake or alcohol consumption over 3 days is not necessarily affected by a higher expected burden in the same way as the preference for the same method. Over a longer time frame, this may be different. Nevertheless, the relatively high willingness to use the food record may have been influenced by a greater familiarity with the method compared to other methods. Food records are similar to popular diet-tracking apps such as MyFitnessPal (50 million downloads for Android in 2017 [24]) and are likely better known to the majority of the participants than the 24-h recall and especially a new, image-based app that requires the self-estimation of the portion size of foods (PortionSize^®^). Furthermore, while willingness differed statistically between several methods, it needs to be acknowledged that the range between the method with the highest willingness (6.6 points for food intake and 6.5 points for alcohol consumption on a 1–8 Likert scale) and the lowest willingness (6.0 points for food intake and 5.7 points for alcohol consumption) was less than 1 point and may be of little practical value. The small difference between methods along with the relatively high willingness ratings across all methods (≥6.0 points for food intake and ≥5.7 points for alcohol consumption) suggests that participants generally seem willing to use all four methods for study/research purposes, even if they rate one method to be relatively more burdensome and less preferred than another. This is encouraging as more frequent engagement with (digital) food logging methods in behavioral interventions has been reported to be associated with greater weight loss over 3 [25] and 12 [26] months. Nonetheless, the actual usage of self-report methods to assess food intake decreases over time, even when efforts are made to reduce burden by, for example, using photographic food records [27].

The findings of the study have implications in clinical research. First, when assessing food/drink intake as an outcome variable in a research study, the same assessment method must be used for all participants. Based on the results of the present study, the demographic characteristics of the study sample (e.g., older vs. younger participants) should be considered when selecting an assessment method since preference and the perceived burden of methods differed by age. This can facilitate the selection of a method that is most preferred by the majority of the study sample. Researchers might also choose to provide standardized training to participants prior to data collection in order to increase comfort with the assessment method; additionally, the need for or the intensity of the training may be influenced by the demographics of the sample. Researchers might also conduct a pilot study or needs assessment in the target population prior to starting a study to determine preferences, feasibility, and training needs. Second, the study has implications in the selection of methods for assessing food/drink intake when delivering an intervention during a clinical study. In this case, it is feasible for different participants to use different methods since the information is primarily used to facilitate intervention delivery and is not used as an outcome variable. Allowing participants to use their preferred method may increase the use of the method over time and further enhance intervention delivery.

A limitation of this analysis is that in the online survey, participants were only presented with descriptions (and illustrations) of the four methods but did not use any of the methods for the suggested period of 3 days to record food intake and alcohol consumption. While the assessment of participants’ general preference and willingness to use certain methods to record food intake and alcohol consumption is important and can inform the choice of method for clinical and study settings, it is conceivable that participants’ preference and willingness to use these methods might change after hands-on use for a given period. However, it has been shown that the participants who tracked their food intake with their preferred method (as indicated by the participants before the intervention) were approximately 50% more adherent to tracking food intake over 12 weeks compared with those who tracked with their non-preferred method [28]. It is therefore unlikely that the real-life use of the methods would change the pattern observed with the RFPM/SmartIntake^®^ as the method has been found to be preferred compared to pen-and-paper records and diet recalls in two studies where participants used the methods [7,9]. Finally, we only included adults residing in the United States in this survey. While adults from all geographic regions in the United States participated (85.8% Louisiana), this limits the generalizability to only similarly developed and high-income nations.

## 5. Conclusions

For both food intake and alcohol consumption, the greatest percentage of participants rated the expected burden of the RFPM/SmartIntake^®^ as low, followed by PortionSize^®^ and food/drink records, and then the 24-h recall. Preference for these methods mirrored the ratings of expected burden, and correlations between low expected burden and high preference were strong for all methods. Because the participants’ preference for a specific method as well as their expected burden of the method likely affect their compliance over time and thereby data quality, our results can be used in conjunction with existing data on the reliability and validity of these methods in order to inform the selection of assessment methods.

## Figures and Tables

**Table 1:** Characteristics of participants who completed the Food Intake Assessment Preference Survey (N = 1959).

Sex, *n* (%)			
	Male	418	(21.3)
	Female	1537	(78.5)
	Other	4	(0.2)
Age Category, *n* (%)			
	<25 years	214	(10.9)
	25–34 years	366	(18.7)
	35–44 years	394	(20.1)
	45–54 years	309	(15.8)
	55–64 years	380	(19.4)
	≥65 years	296	(15.1)
Age (years), mean (SD)		45.9	(16.4)
Race			
	White	1532	(78.2)
	Black	334	(17.0)
	Native American	14	(0.7)
	Asian or Pacific Islander	41	(2.1)
	Other	38	(1.9)
Education, *n* (%)			
	Less than High School	15	(0.8)
	High School or Equivalent	586	(29.9)
	Bachelor’s Degree	680	(34.7)
	Master’s Degree	396	(20.2)
	Doctorate	108	(5.5)
	Other	174	(8.9)
Household Income, *n* (%)			
	<USD 10,000	120	(6.1)
	USD 10,000–50,000	633	(32.3)
	USD 50,000–100,000	666	(34.0)
	USD 100,000–150,000	359	(18.3)
	>USD 150,000	181	(9.2)
Household Food Security, *n* (%) ^a^			
	High Food Security	1450	(74.0)
	Low Food Security	227	(11.6)
	Very Low Food Security	282	(14.4)
Subjective Social Status (1 = lowest, 10 = highest), *n* (%) ^b^			
	10	27	(1.4)
	9	55	(2.8)
	8	175	(8.9)
	7	407	(20.8)
	6	455	(23.2)
	5	408	(20.8)
	4	237	(12.1)
	3	131	(6.7)
	2	41	(2.1)
	1	23	(1.2)
BMI Category, *n* (%) ^c^			
	<25 kg/m^2^	537	(27.4)
	25.0–29.9 kg/m^2^	522	(26.6)
	≥30.0 kg/m^2^	881	(45.0)
BMI (kg/m^2^), mean (SD) ^c^		30.8	(8.8)
Cardiometabolic Diseases ^d^			
	≥1 Disease	830	(42.4)
	No Diseases	1129	(57.6)

^a^ Assessed with the 6-item Short Form of the U.S. Household Food Security Survey Module. ^b^ Assessed with the MacArthur Scale of Subjective Social Status. The scale presets a ‘social ladder’ and asks individuals to place an ‘X’ on the rung (1–10) on which they feel they stand compared to other people in the United States. ^c^ Data available for 1940 of 1959 participants. BMI was calculated from self-reported height and weight. ^d^ Heart disease, type 2 diabetes, hypertension, or dyslipidemia.

**Table 2:** Characteristics of participants who completed the Alcohol Consumption Assessment Preference Survey (N = 466).

Sex, *n* (%)			
	Male	116	(24.9)
	Female	350	(75.1)
Age Category, *n* (%)			
	<25 years	33	(7.1)
	25–34 years	81	(17.4)
	35–44 years	103	(22.1)
	45–54 years	79	(17.0)
	55–64 years	101	(21.7)
	≥65 years	69	(14.8)
Age (years), mean (SD)		45.7	(15.6)
Race			
	White	387	(83.0)
	Black	64	(13.7)
	Native American	2	(0.4)
	Asian or Pacific Islander	6	(1.3)
	Other	7	(1.5)
Education, *n* (%)			
	Less than High School	2	(0.4)
	High School or Equivalent	101	(21.7)
	Bachelor’s Degree	188	(40.3)
	Master’s Degree	110	(23.6)
	Doctorate	30	(6.4)
	Other	35	(7.5)
Household Income, *n* (%)			
	<USD 10,000	18	(3.9)
	USD 10,000–50,000	120	(25.8)
	USD 50,000–100,000	156	(33.5)
	USD 100,000–150,000	108	(23.2)
	>USD 150,000	64	(13.7)
Food Security, *n* (%) ^a^			
	High Food Security	361	(77.5)
	Low Food Security	47	(10.1)
	Very Low Food Security	58	(12.4)
Subjective Social Status (1 = lowest, 10 = highest), *n* (%) ^b^			
	10	4	(0.9)
	9	15	(3.2)
	8	48	(10.3)
	7	109	(23.4)
	6	137	(29.4)
	5	86	(18.5)
	4	46	(9.9)
	3	15	(3.2)
	2	5	(1.1)
	1	1	(0.2)
BMI Category, *n* (%) ^c^			
	<25 kg/m^2^	113	24.2
	25.0–29.9 kg/m^2^	119	25.5
	≥30.0 kg/m^2^	232	49.8
BMI (kg/m^2^), mean (SD) ^c^		31.2	(8.5)
Cardiometabolic Diseases ^d^			
	≥1 disease	203	(43.6)
	No diseases	263	(56.4)
Alcohol consumption history (years), mean (SD)		26.5	(15.9)
Average alcohol consumption frequency, *n* (%)			
	Everyday	24	(5.2)
	3–5 times per week	94	(20.2)
	1–2 times per week	160	(34.3)
	Only on special occasions	188	(40.3)
Number of drinks on typical drinking days, *n* (%) ^e^			
	1 drink	173	(37.1)
	2–3 drinks	230	(49.4)
	3–5 drinks	51	(10.9)
	>5 drinks	12	(2.6)

^a^ Assessed with the 6-item Short Form of the Food Security Survey Module. ^b^ Assessed with the MacArthur Scale of Subjective Social Status. The scale presets a ‘social ladder’ and asks individuals to place an ‘X’ on the rung (1–10) on which they feel they stand compared to other people in the United States. ^c^ Data available for 464 of 466 participants. BMI was calculated from self-reported height and weight. ^d^ Heart disease, type 2 diabetes, hypertension, or dyslipidemia. ^e^ The following examples for a standard drink were given: 12 oz. regular beer, 5 oz. regular wine, 1.5 oz. distilled spirits.

**Table 3:** Preference, expected burden, and willingness to use the four methods of food intake assessment (N = 1959).

**Preference of Methods, *n* (%) ^a^**				
	Food Record			
		Most preferred (first choice)	639	(32.6)
		Second-most preferred (second choice)	301	(15.4)
		Second-least preferred (third choice)	464	(23.7)
		Least preferred (fourth choice)	555	(28.3)
	24-h Recall			
		Most preferred (first choice)	198	(10.1)
		Second-most preferred (second choice)	446	(22.8)
		Second-least preferred (third choice)	592	(30.2)
		Least preferred (fourth choice)	723	(36.9)
	Remote Food Photography Method via SmartIntake^®^ App			
		Most preferred (first choice)	631	(32.2)
		Second-most preferred (second choice)	687	(35.1)
		Second-least preferred (third choice)	530	(27.0)
		Least preferred (fourth choice)	111	(5.7)
	PortionSize^®^			
		Most preferred (first choice)	491	(25.1)
		Second-most preferred (second choice)	525	(26.8)
		Second-least preferred (third choice)	373	(19.0)
		Least preferred (fourth choice)	570	(29.1)
**Expected burden of methods, *n* (%) ^b^**				
	Food Record			
		Least burdensome (first choice)	580	(29.6)
		Second-least burdensome (second choice)	326	(16.6)
		Second-most burdensome (third choice)	477	(24.4)
		Most burdensome (fourth choice)	576	(29.4)
	24-h Recall			
		Least burdensome (first choice)	229	(11.7)
		Second-least burdensome (second choice)	442	(22.6)
		Second-most burdensome (third choice)	578	(29.5)
		Most burdensome (fourth choice)	710	(36.2)
	Remote Food Photography Method via SmartIntake^®^ App			
		Least burdensome (first choice)	686	(35.0)
		Second-least burdensome (second choice)	648	(33.1)
		Second-most burdensome (third choice)	518	(26.4)
		Most burdensome (fourth choice)	107	(5.5)
	PortionSize^®^			
		Least burdensome (first choice)	464	(23.7)
		Second-least burdensome (second choice)	543	(27.7)
		Second-most burdensome (third choice)	386	(19.7)
		Most burdensome (fourth choice)	566	(28.9)
**Willingness to use method over 3 days, mean (SD) ^c^**				
	Food Record		6.6	(2.0)
	24-h Recall		6.1	(2.2)
	Remote Food Photography Method via SmartIntake^®^ App		6.6	(2.0)
	PortionSize^®^		6.0	(2.2)

^a^ Participants were asked to rank the four methods from the most to the least preferred. ^b^ Participants were asked to rank the four methods from the least to the most burdensome. ^c^ Likert Scale: 1 = not at all willing, 8 = very much willing.

**Table 4:** Preference, expected burden, and willingness to use the four methods of alcohol consumption assessment (N = 466).

**Preference of Methods, *n* (%) ^a^**				
	Food Record			
		Most preferred (first choice)	173	(37.1)
		Second-most preferred (second choice)	57	(12.2)
		Second-least preferred (third choice)	128	(27.5)
		Least preferred (fourth choice)	108	(23.2)
	24 h Recall			
		Most preferred (first choice)	54	(11.6)
		Second-most preferred (second choice)	104	(22.3)
		Second-least preferred (third choice)	117	(25.1)
		Least preferred (fourth choice)	191	(41.0)
	Remote Food Photography Method via SmartIntake^®^ App			
		Most preferred (first choice)	108	(23.2)
		Second-most preferred (second choice)	187	(40.1)
		Second-least preferred (third choice)	142	(30.5)
		Least preferred (fourth choice)	29	(6.2)
	PortionSize^®^			
		Most preferred (first choice)	131	(28.1)
		Second-most preferred (second choice)	118	(25.3)
		Second-least preferred (third choice)	79	(17.0)
		Least preferred (fourth choice)	138	(29.6)
**Expected burden of methods, *n* (%) ^b^**				
	Food Record			
		Least burdensome (first choice)	166	(35.6)
		Second-least burdensome (second choice)	64	(13.8)
		Second-most burdensome (third choice)	125	(26.8)
		Most burdensome (fourth choice)	111	(23.8)
	24 h Recall			
		Least burdensome (first choice)	56	(12.0)
		Second-least burdensome (second choice)	106	(22.7)
		Second-most burdensome (third choice)	118	(25.3)
		Most burdensome (fourth choice)	186	(39.9)
	Remote Food Photography Method via SmartIntake^®^ App			
		Least burdensome (first choice)	124	(26.6)
		Second-least burdensome (second choice)	170	(36.5)
		Second-most burdensome (third choice)	140	(30.0)
		Most burdensome (fourth choice)	32	(6.9)
	PortionSize^®^			
		Least burdensome (first choice)	120	(25.8)
		Second-least burdensome (second choice)	126	(27.0)
		Second-most burdensome (third choice)	83	(17.8)
		Most burdensome (fourth choice)	137	(29.4)
**Willingness to use method over 3 days, mean (SD) ^c^**				
	Food Record		6.5	(2.3)
	24 h Recall		5.7	(2.7)
	Remote Food Photography Method via SmartIntake^®^ App		6.4	(2.4)
	PortionSize^®^		6.0	(2.4)

^a^ Participants were asked to rank the four methods from the most to the least preferred. ^b^ Likert Scale: 1 = not at all willing, 8 = very much willing. ^c^ Participants were asked to rank the four methods from the least to the most burdensome.

## Data Availability

The data that support the findings of this study are available from the corresponding author on reasonable request.

## Supplementary Materials

The following are available online at https://www.mdpi.com/article/10.3390/nu13103340/s1, Supplementary Methods: Food Intake Assessment Preference Questionnaire, Alcohol Consumption Assessment Preference Questionnaire; Supplementary Figure S1: Percentage of participants who rated each method as their preferred method of food intake assessment in those <25 years (Panel A), 25–34 years (Panel B), 35–44 years (Panel C), 45–54 years (Panel D), 55–64 years (Panel E), and ≥65 years of age (Panel F). * Denotes a significant difference in ‘not preferred’ for the respective method (*p* < 0.001) in the same panel. Letters (a–d) that differ from each other indicate differences between methods (*p* < 0.001). RFPM, Remote Food Photography Method.
