# Supplementary material for: Preference, Expected Burden, and Willingness to Use Digital and Traditional Methods to Assess Food and Alcohol Intake
Source: Nutrients. 2021 Sep 24;13(10):3340. doi: 10.3390/nu13103340 (PMC8539386; doi:10.3390/nu13103340)
Supplement: Supplementary file 1 [file nutrients-13-03340-s001.zip › Supplementary Figure 1.pdf]

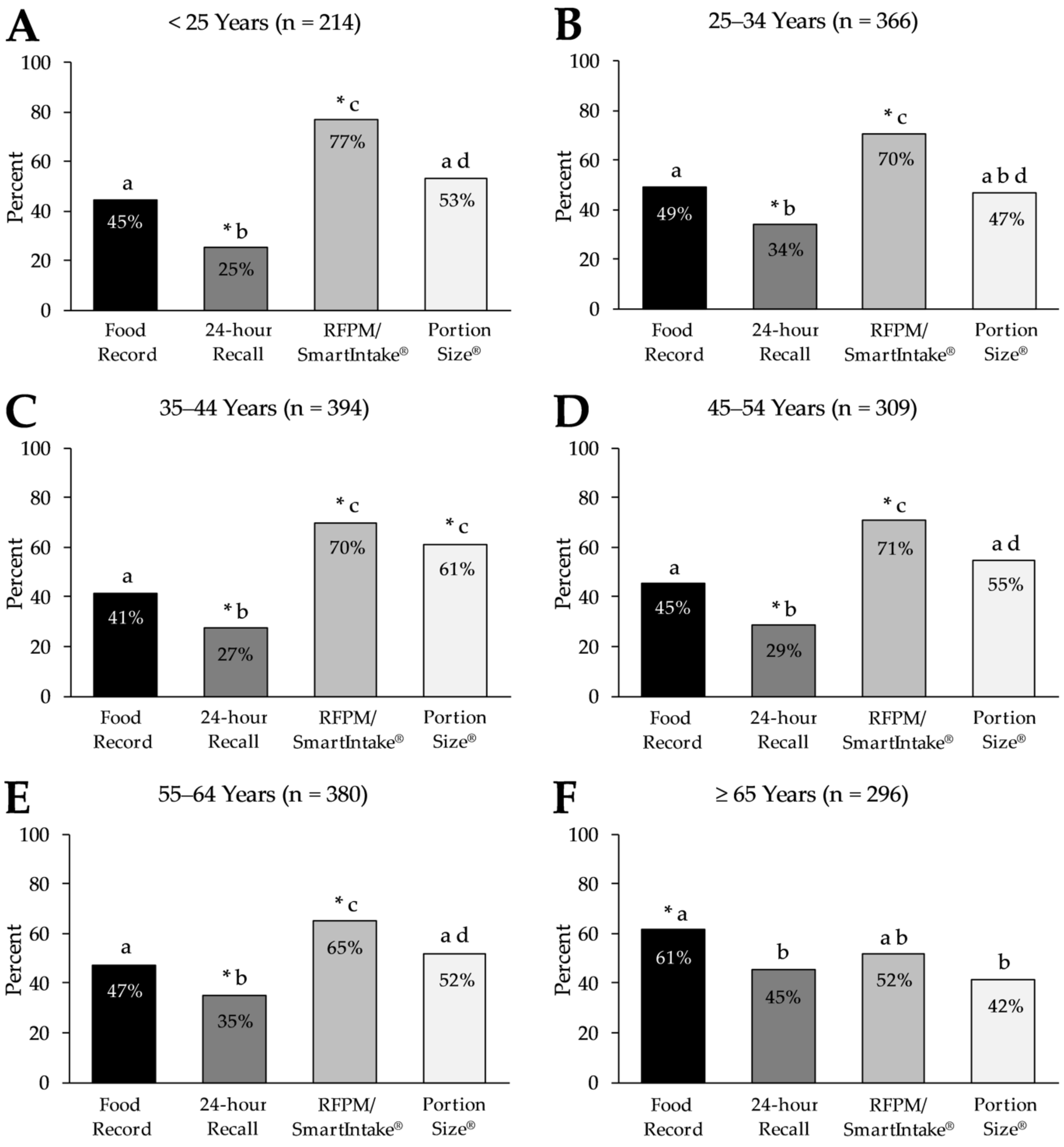

Supplementary Figure 1. Percentage of participants who rated each method as their preferred method of food intake assessment in those <25 years (Panel A), 25–34 years (Panel B), 35–44 years (Panel C), 45–54 years (Panel D), 55–64 years (Panel E), and ≥65 years of age (Panel F). * Denotes a significant difference from 'not preferred' for the respective method ($p < 0.001$) in the same panel. Letters (a–d) that differ from each other indicate differences between methods ($p < 0.001$). RFPM Remote, Food Photography Method.
