# Supplementary material for: Preference, Expected Burden, and Willingness to Use Digital and Traditional Methods to Assess Food and Alcohol Intake
Source: Nutrients. 2021 Sep 24;13(10):3340. doi: 10.3390/nu13103340 (PMC8539386; doi:10.3390/nu13103340)
Supplement: Supplementary file 1 [file nutrients-13-03340-s001.zip › Supplementary Methods.pdf]

SUPPLEMENTARY METHODS

Food Intake Assessment Preference Questionnaire

Thank you for completing this survey. The survey asks you questions about your willingness to use different methods to monitor your food intake and eating behavior in your daily life.

INSTRUCTIONS: Please answer the questions below by filling in the box which best applies to you.

1. **Food records** are a way to record all of the foods and beverages that you consume. You are usually asked to keep these records for 3–7 days. During this period, you would need to carry the record with you and record all foods and beverages that you consume right when you eat or drink them. The food record can be a paper form that you complete by hand. Other ways to keep these records include using a smartphone to complete the record electronically. To increase the accuracy of the record, you need to carefully estimate or weigh how much food you eat, and how many beverages you drink and record those amounts. Additionally, you need to record details about the food or beverage. Those details include things like what cut of meat you are eating, what condiments you added, and how much condiments you added. Finally, you need to record how the food was cooked or prepared. For example, if the food was fried, baked, sautéed, etc.

	**Not at all**							**Very much**
a) How willing are you to use a food diary to monitor your eating and drinking behavior over a period of 3 days?	☐	☐	☐	☐	☐	☐	☐	☐

2. **A 24-hour recall** is another method to track your food and beverage intake. This method is like an interview that is usually conducted via phone or in person. Each interview takes 20 to 30 minutes. You would be asked to recall all of the foods and beverages that you consumed over the previous 24 hours. You also would need to recall and report how much of each food and beverage you consumed. Finally, you would need to recall and report how the foods were prepared (fried, baked, etc.). Because our food intake varies from day to day, you would typically be asked to complete about 3 of these interviews.

	**Not at all**							**Very much**
a) How willing are you to complete three separate 24-hour recall interviews to monitor your eating behavior?	☐	☐	☐	☐	☐	☐	☐	☐

3. Smartphone-based methods can **record food intake based on pictures of food** that you capture with a smartphone app. Specifically, you would use an app (SmartIntake®) to take pictures of your meals before and after you eat. If it is not clear what you are eating or drinking, you would type in a brief description of those foods. The app then automatically sends the pictures and information you entered to nutrition professionals. Those nutrition professionals can then estimate how much you ate and drank based on the pictures. The app also automatically reminds you to capture images of your meals. Those reminders are customized based on your schedule and eating habits. Sample pictures that were taken before and after a snack are provided below.

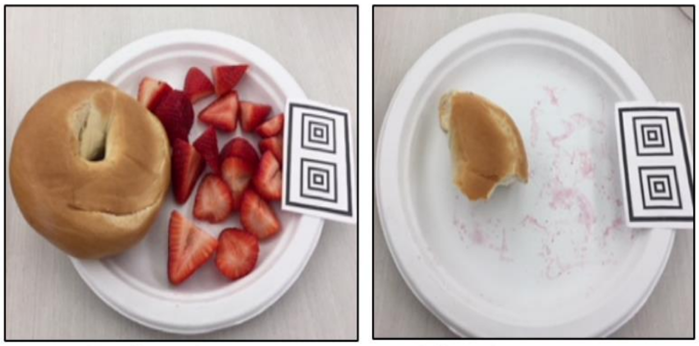

Before image. *After image.*

Description: "Plain bagel and strawberries"

Fazzino et al. JMIR Mhealth Uhealth; 2018; 6(9):e10460

	**Not at all**							**Very much**
a) How willing are you to use this method to monitor your eating behavior over a period of 3 days?	☐	☐	☐	☐	☐	☐	☐	☐

4. More recently, smartphone apps have been developed that do not require the analysis of the food images by a nutrition professional. Rather, you would **estimate the portion size of the foods directly in the app**. You would take a picture of your meal before you ate. You would then identify the foods and beverages in the meal via a drop-down list or with a search function. To estimate the portion size of the foods in the picture, you would do one of two things. First, you can enter in the amount of foods consumed or the size of the food if it is known (eg, 4 Famous Amos cookies, one 12-ounce soda). Second, you can use templates that appear in the app. You can adjust the size of these templates and move them within the picture of the foods. Hence, to estimate portion size, you would change the size of the template and move it so it covers the food. There is an example of this in the picture below, where a template that looks like a deck of cards was placed over scrambled eggs. The app then uses this information to automatically and immediately estimate how much food is on your plate. After the meal, you enter if you ate everything, left a certain amount of the food on your plate, or you can use the templates again to estimate large portions of leftovers. This allows the app to provide a more accurate estimate of how much you ate. The app gives you real-time feedback about how many calories you ate and the nutrient composition of your meal and overall diet. An example of that feedback is provided in the picture below.

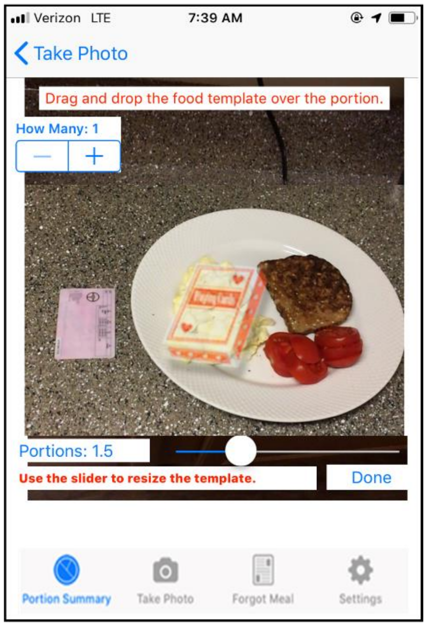

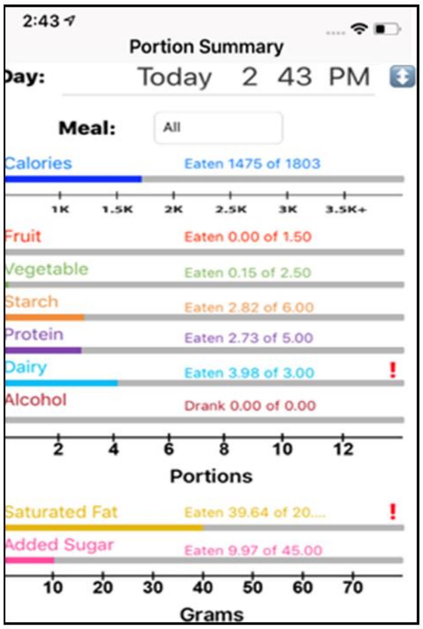

	**Not at all**							**Very much**
a) How willing are you to use this method to monitor your eating behavior over a period of 3 days?	☐	☐	☐	☐	☐	☐	☐	☐

5. In the previous questions, you were presented with the following 4 methods to track food intake.

**Food Record:**	Record all foods and beverages (including portion size and how the foods were prepared) right when you eat or drink them over a period of 3-7 days. These records or diaries can be pen-and-paper, or electronic.
**24-hour Recall:**	Interview (20-30 minutes), during which you are asked to recall all of the foods and beverages that you consumed over the previous 24 hours. Typically, 3 of these interviews are conducted.
**Remote Food Photography Method via SmartIntake app:**	Take pictures of your meals before and after you eat via a smartphone app. The pictures are then sent to nutrition professionals for analysis.
**Smartphone photography with in-app estimation of portion size (PortionSize®):**	Take pictures of your meals before and after you eat via a smartphone app and enter in the amount of countable foods or use templates that appear in the app to estimate your portion size or.

a) Assume that you will need to record your food and beverage intake for 3 days. Which of the four previously described methods sounds **<u>the least burdensome</u>**? Please rate each method from least to most burdensome. Only one selection per method is allowed.

	**First Choice (least burdensome)**	**Second Choice**	**Third Choice**	**Fourth Choice (most burdensome)**
**Food Record:**	☐	☐	☐	☐
**24-hour Recall:**	☐	☐	☐	☐
**Remote Food Photography Method via SmartIntake app:**	☐	☐	☐	☐
**Smartphone photography with in-app estimation of portion size (PortionSize®):**	☐	☐	☐	☐

b) Assume that you will need to record your food and beverage intake for 3 days. Which of the four previously described methods would you **<u>prefer to use</u>**? Please rate each method from least to most preferred. Only one selection per method is allowed.

	First Choice (most preferred)	Second Choice	Third Choice	Fourth Choice (least preferred)
**Food Record:**	☐	☐	☐	☐
**24-hour Recall:**	☐	☐	☐	☐
**Remote Food Photography Method via SmartIntake app:**	☐	☐	☐	☐
**Smartphone photography with in-app estimation of portion size (PortionSize®):**	☐	☐	☐	☐

Alcohol Consumption Assessment Preference Questionnaire

Thank you for completing this survey. The survey asks you questions about your willingness to use different methods to monitor your alcohol consumption in your daily life.

INSTRUCTIONS: Please answer the questions below by filling in the box which best applies to you.

1. **Drink records** are a way to self-record all alcoholic beverages that you consume. You are usually asked to keep these records for 3–7 days. During this period, you would need to carry the record with you and record **all** alcoholic beverages that you consume right when you drink them. The drink record can be a paper form that you complete by hand. Other ways to keep these records include using a smartphone to complete the record electronically. To increase the accuracy of the record, you need to carefully record the type of drink, measure the amount (if possible), and estimate its alcohol content.

	**Not at all**							**Very much**
a) How willing are you to use a drink diary to monitor your drinking behavior over a period of 3 days?	☐	☐	☐	☐	☐	☐	☐	☐

2. A **24-hour recall** is another method to track your alcoholic beverage consumption. This method is like an interview that is usually conducted via phone or in person. Each interview takes 20 to 30 minutes. You would be asked to recall all alcoholic beverages that you consumed over the previous 24 hours. You also would need to recall and report the alcohol content of each beverage you consumed. Because our drinking behavior varies, you would typically be asked to complete about 3 of these interviews.

	**Not at all**							**Very much**
a) How willing are you to complete three separate 24-hour recall interviews to monitor your drinking behavior?	☐	☐	☐	☐	☐	☐	☐	☐

3. Smartphone-based methods can **record alcohol consumption based on pictures of drinks** that you capture with a smartphone app. Specifically, you would use an app (SmartIntake®) to take pictures of your drink before and after you consume it. If it is not clear what you are drinking, you would type in a brief description of the drink. The app then automatically sends the pictures and information you entered to nutrition professionals. Those nutrition professionals can then estimate how much alcohol you consumed based on the pictures. The app also automatically reminds you to capture images of your alcoholic drinks. Those reminders are customized based on your schedule and drinking habits. Sample pictures that were taken before and after a drink are provided below.

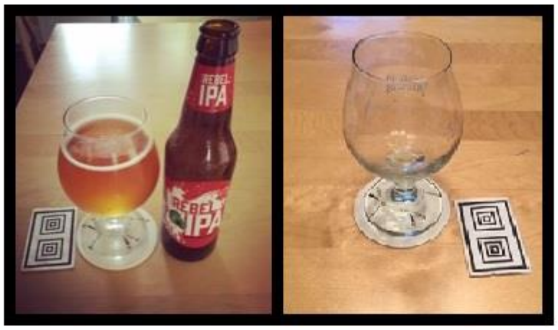

Before image After image

Description: "Rebel IPA Sam Adams"

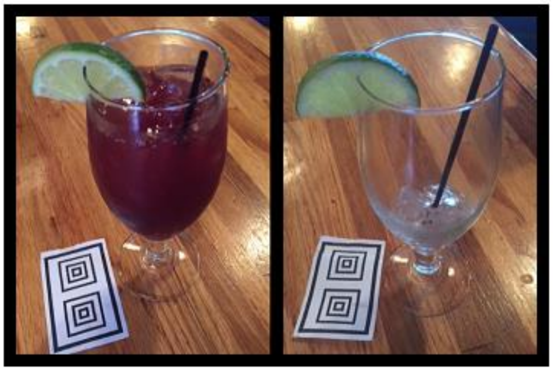

Before image After image

Description: "Sangria with lime"

Fazzino et al. JMIR Mhealth Uhealth; 2018; 6(9):e10460

	**Not at all**							**Very much**
a) How willing are you to use this method to monitor your drinking behavior over a period of 3 days?	☐	☐	☐	☐	☐	☐	☐	☐

4. More recently, smartphone apps have been developed that do not require the analysis of the drink images by a nutrition professional. Rather, you would **estimate the drink size and its alcohol content directly in the app**. You would take a picture of your drink before you consume it. You would then identify the drink or its ingredients via a drop-down list or with a search function. To estimate the size of the drink in the picture, you would do one of two things. First, you can select the size of the drink, for example, 12 ounces. Second, especially for drinks where you do not know the size of the glass, you can use templates that appear in the app. The templates are in the shape of different glasses and you can change the size of these templates to match the size of your drink in the image. After you consumed the drink, you would indicate if you drank all, left a certain amount, or you can use the templates again to estimate leftovers. An example of a picture captured with the app and the template used to estimate the portion size is provided below.

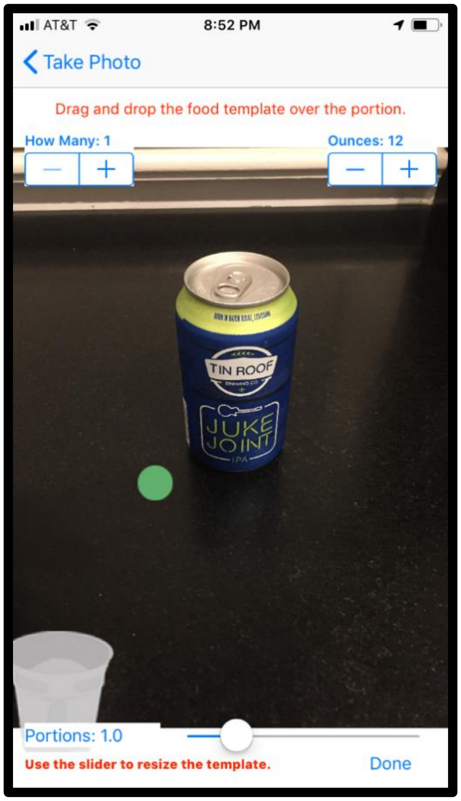

	Not at all							Very much
a) How willing are you to use this method to monitor your drinking behavior over a period of 3 days?	☐	☐	☐	☐	☐	☐	☐	☐

5. In the previous questions, you were presented with the following 4 methods to track drinking behavior.

**Drink Record:**	Record all alcoholic beverages (including amount and alcohol content) right when you drink them over a period of 3-7 days. These records or diaries can be pen-and-paper, or electronic.
**24-hour Recall:**	Interview (20-30 minutes), during which you are asked to recall all of the alcoholic beverages, including their alcohol content, that you consumed over the previous 24 hours. Typically, 3 of these interviews are conducted.
**Remote Food Photography Method via SmartIntake app:**	Take pictures of your alcoholic beverages before and after you consume them via a smartphone app. The pictures are then sent to nutrition professionals for analysis.
**Smartphone photography with in-app estimation of portion size (PortionSize®):**	Take pictures of your alcoholic beverages before and after you consume them via a smartphone app. Enter the drink size in the app, or use templates to estimate the size of your drink.

a) Assume that you will need to record your alcoholic beverage intake for 3 days. Which of the four previously described methods sounds **<u>the least burdensome</u>**? Please rate each method from least to most burdensome. Only one selection per method is allowed.

	**First Choice (least burdensome)**	**Second Choice**	**Third Choice**	**Fourth Choice (most burdensome)**
**Drink Records:**	☐	☐	☐	☐
**24-hour Recall:**	☐	☐	☐	☐
**Remote Food Photography Method via SmartIntake app:**	☐	☐	☐	☐
**Smartphone photography with in-app estimation of portion size (PortionSize®):**	☐	☐	☐	☐

b) Assume that you will need to record your alcoholic beverage intake for 3 days. Which of the four previously described methods would you **prefer to use**? Please rate each method from least to most preferred. Only one selection per method is allowed.

	First Choice (most preferred)	Second Choice	Third Choice	Fourth Choice (least preferred)
**Drink Records:**	☐	☐	☐	☐
**24-hour Recall:**	☐	☐	☐	☐
**Remote Food Photography Method via SmartIntake app:**	☐	☐	☐	☐
**Smartphone photography with in-app estimation of portion size (PortionSize®):**	☐	☐	☐	☐
